# Dopamine and α-synuclein dysfunction in Smad3 null mice

**DOI:** 10.1186/1750-1326-6-72

**Published:** 2011-10-13

**Authors:** Silvia Tapia-González, Rosa M Giráldez-Pérez, M Isabel Cuartero, M José Casarejos, M Ángeles Mena, Xiao-Fan Wang, Amelia Sánchez-Capelo

**Affiliations:** 1 Departamento de Neurobiología-Investigación, Hospital Ramón y Cajal, IRYCIS, Madrid, Spain; 2 Department of Pharmacology and Cancer Biology, Duke University Medical Center, Durham, North Carolina, USA; 3 Centro de Investigaciones Biomédicas en Red - Enfermedades Neurodegenerativas (CIBERNED), Spain

**Keywords:** Smad3, Parkinson's disease, Synucleinopathy, Dopamine, Astrocytes, TGF-β, α-Synuclein, MAO-B, MAPK

## Abstract

**Background:**

Parkinson's disease (PD) is characterized by dopaminergic neurodegeneration in the substantia nigra (SN). Transforming growth factor-β1 (TGF-β1) levels increase in patients with PD, although the effects of this increment remain unclear. We have examined the mesostriatal system in adult mice deficient in Smad3, a molecule involved in the intracellular TGF-β1 signalling cascade.

**Results:**

Striatal monoamine oxidase (MAO)-mediated dopamine (DA) catabolism to 3,4-dihydroxyphenylacetic acid (DOPAC) is strongly increased, promoting oxidative stress that is reflected by an increase in glutathione levels. Fewer astrocytes are detected in the ventral midbrain (VM) and striatal matrix, suggesting decreased trophic support to dopaminergic neurons. The SN of these mice has dopaminergic neuronal degeneration in its rostral portion, and the pro-survival Erk1/2 signalling is diminished in nigra dopaminergic neurons, not associated with alterations to p-JNK or p-p38. Furthermore, inclusions of α-synuclein are evident in selected brain areas, both in the perikaryon (SN and paralemniscal nucleus) or neurites (motor and cingulate cortices, striatum and spinal cord). Interestingly, these α-synuclein deposits are detected with ubiquitin and P^S129^-α-synuclein in a core/halo cellular distribution, which resemble those observed in human Lewy bodies (LB).

**Conclusions:**

Smad3 deficiency promotes strong catabolism of DA in the striatum (ST), decrease trophic and astrocytic support to dopaminergic neurons and may induce α-synuclein aggregation, which may be related to early parkinsonism. These data underline a role for Smad3 in α-synuclein and DA homeostasis, and suggest that modulatory molecules of this signalling pathway should be evaluated as possible neuroprotective agents.

## Background

PD is characterized by the progressive degeneration of dopaminergic neurons in the SN *pars compacta*. These neurons project to the ST and secrete DA in order to control voluntary movements and rewarding events [1]. Another significant feature of PD is the presence of cytoplasmic and neuritic inclusions of α-synuclein, known as LB and neurites. The aetiology of idiopathic PD is unknown, although current hypotheses focus on increased oxidative stress, aberrant protein folding, defective proteasome degradation or mitochondrial dysfunction. Given this diversity in the underlying molecular mechanisms, it has been suggested that multiple factors may cause the disease [2]. However, the central mechanisms that induce dopaminergic neuron loss and α-synuclein aggregation remain unclear.

TGF-β1 is a cytokine that might mediate some molecular mechanisms of the disease. This molecule is expressed at low levels in uninjured brains, while it is up-regulated in the brain in association with diseases such as Parkinson's and Alzheimer's disease, Down syndrome, ischemic lesion, hydrocephalus or spinal cord injury [3-10].

TGF-β1 is a highly pleiotropic molecule that can regulate cell proliferation, migration, differentiation and apoptosis. Its effect is cell type and context-dependent, and it may provide signals for both cell survival and apoptosis [11]. Moreover, a role for TGF-β in neuronal plasticity is becoming evident. In Drosophila, the maintenance and specialization of synapses at the neuromuscular junction requires TGF-β-mediated transcriptional regulation [12,13]. In Aplysia, TGF-β1 induces long-term facilitation in sensory-motor synapses [14].

During mammalian embryonic development, TGF-β3 (but not TGF-β1) is necessary for the survival of midbrain dopaminergic neurons at perinatal stages [15]. Hence, while TGF-β3 appears to exert its effects on newborns neurons, TGF-β1 might have pathological effects in adults. The context-dependent effects of these factors are clearly observed in sensory neurons, where ontogenic neuronal death is increased by TGF-β [16]. In the nigrostriatal system of MPTP-treated mice, adenoviral overexpression of active TGF-β1 produces a decrease in the survival of dopaminergic neurons, accompanied by higher levels of striatal DA depletion [17,18]. Despite these studies, the role of TGF-β in the adult mesostriatal system is not well characterized.

The TGF-β subfamily of cytokines exert their effect by binding to heteromeric receptor complexes at the cell surface that contain a type I (primarily ALK5, but also ALK2 or ALK1) and a type II receptor (TβR-II or ActR-IIB). Ligand binding to the receptor complex promotes the recruitment and phosphorylation of Smad3 and/or Smad2. Activated Smad3/2 interacts with Smad4 inducing their translocation to the nucleus, where they bind to DNA through specific Smad-binding elements, thereby promoting targeted gene transcription. Some of the ligands that can activate Smad3 and/or Smad2 are TGF-β1, -β2, -β3, Inhibin, Activin, Nodal, GDF1, Vg1, and Lefty. This canonical TGF-β pathway can cross-talk at multiple levels with other signalling pathways such as those mediated by MAPK, Fas, CaMKII, PKC, etc [11].

To address the role of TGF-β in the adult nigrostriatal system, we have analyzed Smad3 null mice. We provide evidence that Smad3 deficiency diminishes the trophic and astrocytic support to nigral dopaminergic neurons, as well as strongly altering DA metabolism. In addition α-synuclein over-expression is produced in these mice, with the formation of aggregates in different telencephalic, mesencephalic and romboencephalic regions.

## Results

### Smad3 is expressed in dopaminergic neurons of the SN

**Figure 1:**
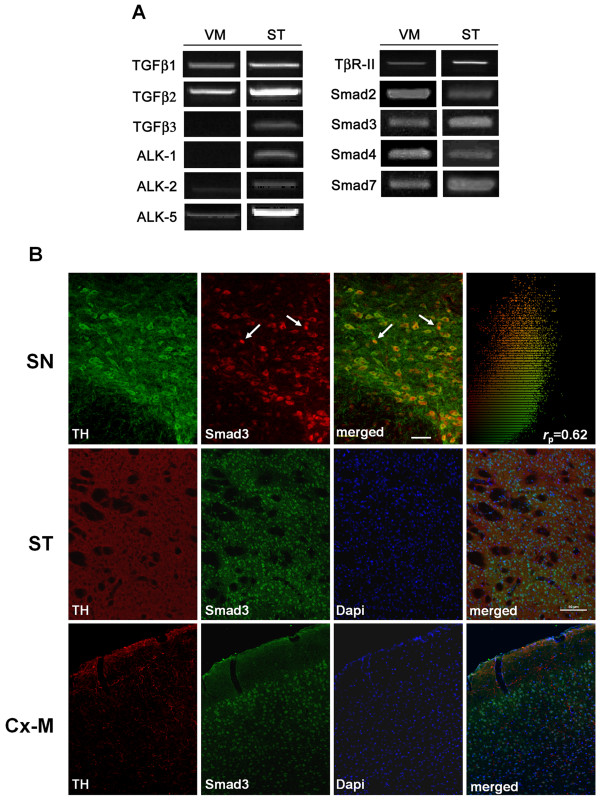
**Smad3 is expressed in dopaminergic neurons of the SN**. (A) Expression of TGFβ ligands, receptors and Smad3 signalling molecules in the VM and ST of adult mice. RT-PCR from three adult mice are shown under basal conditions for the expression of TGF-β1, -β2, and -β3 ligands, ALK1, ALK2, ALK5 type I receptors, TβR-II type II receptor, Smad2 and Smad3 receptor activated Smads, the common Smad4, and the inhibitory Smad7. Every PCR reaction was performed using 1 μl of the cDNA pool, except for TGF-β3 and ALK1 in the VM where 1, 2 and 3 μl were tested. (B) Coronal sections were double immunostained for TH and Smad3. SN dopaminergic neurons express Smad3 in the cytoplasm, although some neurons have Smad3 in the nucleus (arrows). Quantitative estimate of the values representing colocalized pixels in the dual-color image shows the co-localization of both signals in the SN (Pearson correlation coefficient = 0.62). Confocal images of TH/Smad3 double-labelling in the ST and the motor cortex (Cx-M) suggested region co-localization, but not at the cellular level. Scale bar 50 μm.

**Figure 2:**
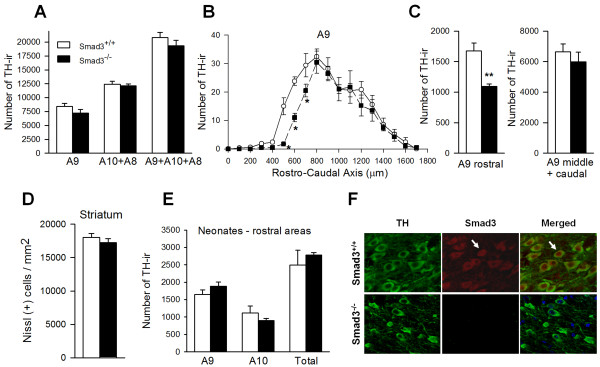
**Degeneration of dopaminergic neurons in the rostral SN of Smad3 null mice**. (A) Quantification of TH-ir neurons was determined using unbiased stereological methods in the midbrain A9, A10 and A8 dopaminergic areas (which include the SN, ventral tegmental area and retrorubral field, respectively). No clear differences were observed between mice when the entire pool of TH-ir cells was counted in these areas. The stereological counting method had a CE < 0.07 for each mouse. (B-C) Representation of the SN (A9) along the rostro-caudal axis showed that Smad3^-/- ^mutant mice had fewer TH-ir neurons than Smad3^+/+ ^in the initial rostral 500 μm of the SN (*P < 0.05, **P < 0.01, Mann-Whitney Rank Sum test). Similar values were detected between mice in the middle and caudal regions. Unpaired *t*-test, n = 5 for Smad3^+/+ ^and n = 6 for Smad3^-/- ^mice. (D) Unbiased stereological quantification of Nissl(+) neurons in the ST of Smad3^+/+ ^and Smad3^-/- ^mice (Unpaired *t*-test, n = 4 mice per genotype). CE ≤ 0.05. (E) Quantification of TH-ir neurons in the rostral portion of the midbrain at perinatal stage (P0). Similar values were found between Smad3^+/+ ^and Smad3^-/- ^mice, indicating no developmental defect, but a postnatal degenerative process in Smad3 deficiency (Student's *t*-test, n = 3-4 per genotype). (F) Confocal microscopic images of Smad3/TH double-labelled neurons in the SN of Smad3^+/+ ^and Smad3^-/- ^mice. In control tissues, some neurons show Smad3 translocated to the nucleus (arrow). Smad3 null mutant mice have no expression of the protein.

To determine whether molecules related to TGF-β signalling are expressed in the adult murine nigrostriatal system, we examined the expression of mRNA transcripts encoding: TGF-β1, -β2 and -β3 ligands; type I receptors ALK1, ALK2 and ALK5; the type II receptor TβR-II; the Smad signalling molecules Smad2, Smad3, Smad4; and the inhibitor Smad7. RT-PCR analysis showed that all these molecules are expressed in the VM and ST of adult mice, with the exception of TGF-β3 and AKL1 in the VM (Figure 1A). The absence of TGF-β3 expression in the VM suggests that this molecule is not critical for Smad3 signalling in adults, in contrast with perinatal stages [15]. We further assessed whether TGF-β signalling might be present in dopaminergic neurons by histological analysis of Smad3 on tyrosine hydroxylase (TH) positive neurons. Dopaminergic neurons in the SN express Smad3, primarily in the cytoplasm, although some neurons with nuclear localization were also detected (Figure 1B, 2F, arrows). We also observed a population of TH^+^/Smad3^- ^and Smad3^+^/TH^- ^cells in the SN. The presence of Smad3 in TH-ir cells indicates that TGF-β ligands can act directly on dopaminergic neurons. We further assessed Smad3 expression in other brain areas related to parkinsonism, such as the ST and the motor cortex. Confocal analysis suggested that Smad3 was present in the nucleus of cells of the striatal matrix compartment (the TH-fibre rich region). On the other hand, TH-fibre innervation was detected throughout the motor cortex, with Smad3 mainly expressed in the nucleus of cells of the third cortical layer (Figure 1B).

### Smad3 depletion leads to fewer dopaminergic neurons in the rostral SN

Because TGF-β1 can provide both pro-survival or death signals to cells, we determined whether Smad3 deficiency altered the number of dopaminergic neurons in the VM. We analyzed the nigrostriatal system in 2- to 3 -month-old Smad3 knock-out male mice in the basal state. These mice develop to term and live to adulthood, surviving to 2 to 4 months of age. They exhibited subtle phenotypic abnormalities, such as osteopenia, while 1/3 of the mice developed malformations of the forelimbs and rib cage [19], curvature of the spine (kyphosis) and resting tremor (data not shown). The brains of Smad3^-/- ^mice exhibited a grossly normal morphology. Indeed, when we used unbiased stereological methods to quantify the number of dopaminergic neurons along the rostro-caudal axis of the A9, A10 and A8 areas of Smad3^-/- ^and Smad3^+/+ ^mice, the numbers were similar in the null and wild-type mice genotypes in both the A10 and A8 dopaminergic areas (Figure 2A, Additional file 1A). However, Smad3 depletion led to a 34.8% decrease in the number of TH-ir neurons in the rostral SN (P = 0.004, Figure 2B-C). We could not detect stronger Smad3 expression in the rostral portion of the SN (data not shown), suggesting a functional spatial differentiation of this signalling molecule. Although the number of dopaminergic neurons decreased in the rostral portion of SN in Smad3 null mice, TH protein levels in the VM were similar to those found in control mice (P = 0.566, data not shown). We assessed apoptosis in the SN of these mice using TUNEL assay (to measure nuclear DNA fragmentation) and morphological criteria (shrunken, smooth-appearing nuclei that have lost the granularity, associated with nuclear chromatin condensation) [20]. We could not detect a clear increase in apoptotic cells in the SN of Smad3^-/- ^mice (Additional file 1B). Considering that the criteria used to measure apoptosis last for a few hours (both nuclear condensation and fragmentation), it seemed possible that neuronal death might be greater at older ages. Unfortunately, these knockout mice did not survive beyond 4 months of age and thus, an analysis of older animals was not possible.

TGF-β3, but not TGF-β1, has been shown to be required for the survival of dopaminergic neurons in perinatal stages [15]. In order to discriminate between a developmental defect and a degenerative process in the SN of adult Smad3 null mice, we quantified the number of rostral TH-ir neurons in day 0 of postnatal age (P0). Similar number of dopaminergic neurons was found in Smad3 null mice and control mice (Figure 2E), indicating a postnatal degenerative process in Smad3 deficiency (Figure 2C).

We evaluated whether Smad3 was necessary to maintain dopaminergic striatal innervation from nigral dopaminergic neurons, and whether the rostral deficit in DA neurons might have a positional effect in the ST. We quantified the optical density of TH-ir fibres along the rostro-caudal axis, as well as in the dorsal and ventral ST. No changes were observed between the wild type and mutant mice (Additional file 2A). Furthermore, there was no alteration in striatal TH protein content (Additional file 2B). Dopamine transporter (DAT) immunoreactivity was also assayed in the ST of these mice. Smad3 deficiency resulted in a not-statistically significant trend to decrease DAT expression in the rostral ST (P = 0.095, Additional file 3A-C). This trend is observed in the rostral ST, similar to the rostral decrease on TH-ir neurons in the SN, although in a wider area. We further analyzed whether there are any neuronal loss in the ST. Unbiased stereological quantification of Nissl(+) neurons showed that Smad3 deficiency did not alter the number of striatal neurons (Figure 2D), suggesting that the pro-survival effect of Smad3 may be specific to the dopaminergic neurons of the SN in the mesostriatal system. Together, the loss of TH-ir neurons in the SN and the trend to decrease the presynaptic DA marker in the ST (DAT) seems to suggest an early loss of dopaminergic neurons in Smad3 null mice.

### Smad3 null mice have less GFAP expressing astrocytes

**Figure 3:**
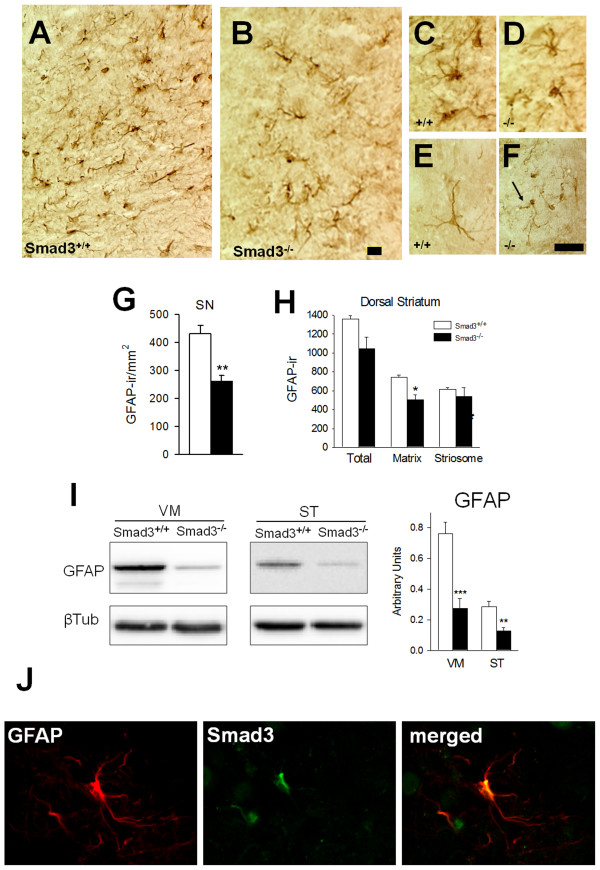
**Smad3^-/- ^mice have fewer nigrostriatal astrocytes**. In the SN the number of GFAP-ir astrocytes decreased in Smad3 deficiency (A-B, G). Illustrations depict non-activated astrocytes as verified by the labelling of thin, elongated processes in the wild-type (C) and null (D) mice. In the ST, atrophic astrocytes could be detected in Smad3 ^-/- ^null mice (F, arrow) when compared to Smad3^+/+ ^mice (E). (H) Number of GFAP-ir cells in the striatal matrix and striosomes. The figure represents the GFAP-ir cells per mm^2 ^in one striatal hemisphere. (I) The GFAP protein content is decreased in both the VM and ST of Smad3 null mice (Student's *t*-test, n = 4-6 per genotype) (J) Confocal images of Smad3 and GFAP double-labelled cells in the SN of wild-type mice showed co-localization of Smad3 in astrocytes. *P < 0.05, **P < 0.01, ***P < 0.001

The effect of the absence of Smad3 on astrocytes -expressing glial fibrillary acidic protein (GFAP)- and microglia -detected by CD11b-ir, which is upregulated on activated microglia- was explored in the nigrostriatal system of these mice. Microglial cells were not clearly altered in Smad3 deficient mice (data not shown). However, the number of astrocytes in the SN apparently decreased even when assessed at low magnification (Figure 3A-B). Quantification of the ventral, middle and lateral SN (Additional file 4A-C) showed that Smad3^-/- ^mice had 38.0% less GFAP-ir cells than Smad3^+/+ ^mice (P = 0.004) (Figure 3G). Indeed, in the ST, Smad3 deficiency induces a 32.1% decrease in GFAP-ir cells in the matrix compartment of the dorsal zone (the TH-fibre rich region; P = 0.014, Figure 3H). To further explore this effect we examined the levels of GFAP protein expressed in the VM and ST of these animals. The GFAP protein content is decreased in null mutant mice by 63.9% in the VM (P < 0.001) and by 55.3% in the ST (P = 0.004, Figure 3I). Moreover, while morphological changes were not apparent in astrocytes from the SN (Figure 3A-D), some atrophy in the arborization of astrocytes, with thin branching, is exhibited in the ST of Smad3 null mice (Figure 3E-F).

We searched for Smad3 immunoreactivity in GFAP(+) astrocytes within the SN of wild-type mice. Co-localization of Smad3 and GFAP was evident (Figure 3J), suggesting that Smad3 could provide a survival signalling for astrocytes. Astrocytes are the main element of brain homeostatic system and loss of astrocytes in Smad3 deficient mice may decrease trophic and metabolic support to surrounding neurons.

### Striatal DA catabolism and oxidative stress increase when Smad3 is depleted

**Figure 4:**
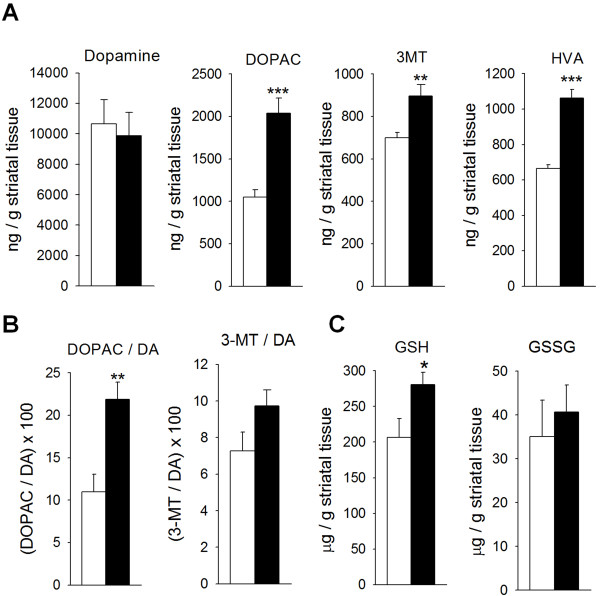
**Smad3 ablation induces an increase in MAO-mediated DA catabolism in the ST**. (A) Loss of Smad3 leads to increased catabolism of striatal DA, with no alterations in total neurotransmitter content. (B) The catabolism of DA in Smad3 deficient mice is primarily MAO-mediated (DOPAC/DA ratio) as compared to COMT (3-MT/DA). (C) The striatal GSH content of Smad3 ^-/- ^mice is also higher than in Smad3 ^+/+ ^mice. (*P < 0.05, **P < 0.01, ***P < 0.001, Student's *t*-test, n = 6 per genotype)

We further tested whether striatal DA levels is altered. Similar levels of DA were found in Smad3 null mice and control mice, although a strong increase in DA catabolism was detected in the former (Figure 4A). Thus, in Smad3 deficient mice the striatal levels of DOPAC, homovanillic acid (HVA), and 3-methoxy-tyramine (3-MT) increased by 94.5% (P = 0.001), 59.8% (P < 0.001), and 28.1% (P = 0.007), respectively. The increase in DA oxidation was primarily mediated by MAO since the DOPAC/DA ratio augmented 98.8% (P = 0.004, Figure 4B). Catechol-O-methyl transferase (COMT) activity, quantified by 3-MT/DA ratio, did not increase significantly (33.9%, P = 0.101). This Smad3-dependent effect might be relatively specific for DA because striatal serotonin was not clearly altered (Additional file 5). It is known that the oxidation of DA to DOPAC is accompanied by the stoichiometric reduction of oxygen to H_2_O_2 _[21]. Hence, the 2-fold increment in MAO-mediated DA oxidation must increase striatal oxidative stress. The normal detoxification pathway for H_2_O_2 _is the glutathione system, supplied by the mitochondria. We measured striatal glutathione content in its reduced (GSH) and oxidized (GSSG) forms (Figure 4C). Smad3^-/- ^mice had 35.9% more striatal GSH than Smad3^+/+ ^mice (P = 0.043). We could speculate on a compensatory mechanism by anti-oxidant GSH defence in order to detoxify excess of H_2_O_2 _produced by increased DA turnover in Smad3 deficiency.

### MAO-B increases in the ventral midbrain of Smad3 null mice

**Figure 5:**
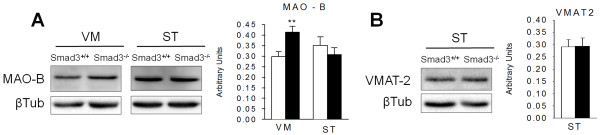
**Smad3 deficiency leads to increased MAO-B levels in the ventral midbrain**. (A) Smad3^-/- ^mice had higher MAO-B levels in the VM than Smad3^+/+ ^mice. (B) VMAT-2 protein contents are not altered in Smad3^-/- ^mice. Bars represent values normalized to β-tubulin from 6 mice per group, each measured two to three times. **P < 0.01, Student's *t- *test.

The increment in DA catabolism without a modification in DA levels may be related to alterations in different mechanisms, such as increased MAO levels or decreased vesicular DA storage. Once re-uptaked from the presynaptic terminals, DA can either be repackaged into synaptic vesicles by the vesicular monoamine transporter (VMAT-2) or catabolyzed by MAO. We analyzed the striatal VMAT-2 content and we observed that Smad3 deficiency did not alter their protein levels (Figure 5B). However, Smad3 ablation increased the MAO-B content in the VM by 38.9% (P = 0.010, Figure 5A). In the ST, the change in MAO-mediated DA catabolism (Figure 4B) does not seem to correlate with protein content alteration, suggesting changes in MAO-B activity for the 2-fold increment in DOPAC/DA ratio. On the other hand, dopaminergic somatodendrites in SN can synthesize, store and release DA. The increment in MAO-B protein levels could participate in the catabolism of DA in somatodendrites. Moreover, the SN contains high levels of MAO-B positive astrocytes and its elevation in mouse brain results in parkinsonism [22]. Moreover, MAO-B activity levels have been found to be doubled in the SN of PD [23].

### Smad3 deficiency decreases the phosphorylation of the Smad2 linker region

**Figure 6:**
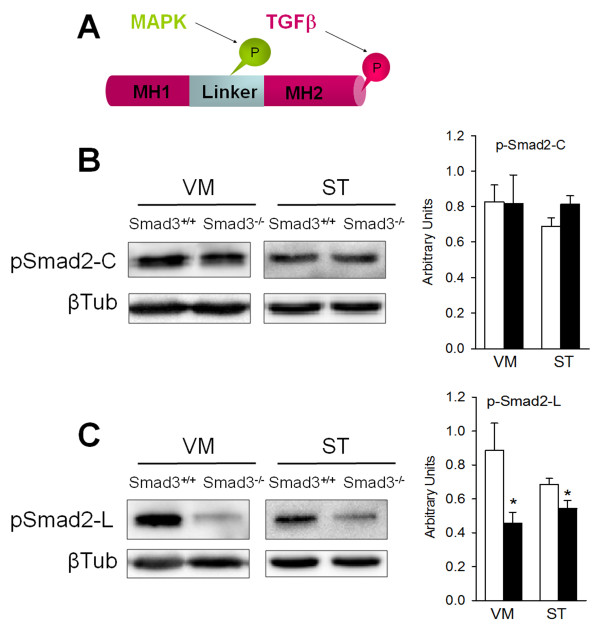
**Decreased Smad2 phosphorylation in Smad3 null mice**. (A) The MH1 and MH2 domains in Smad2, conserved in other Smads, are connected by a less-conserved linker region. TGF-β and MAPKs signalling induces phosphorylation of certain sites in Smad2. (B) The level of activated Smad2 (carboxy-terminal phosphorylation) is not modified by Smad3 ablation. (C) However, in Smad3 null mice the linker region is less phosphorylated, suggesting a decrease in an intracellular regulatory signal of Smad2 (*P < 0.05, Student's *t*-test, n = 6 per genotype, each measured two to three times).

Smad3-deficiency may induce an increment in Smad2 signalling in an attempt to compensate for the TGF-β signalling deficit. In order to evaluate this possibility we measured the phosphorylation of Smad2 at two different locations (Figure 6A). The carboxyl terminus is phosphorylated by TGF-β activated type I receptors, while the linker region is phosphorylated by regulatory intracellular signals such as MAPKs, CaMKII or CDKs [24]. In immunoblots, phosphorylation of the Smad2 carboxy-terminal was not altered in the VM or the ST of Smad3^-/- ^mice (Figure 6B). However, the phosphorylation of the linker region decreased by 48.4% in the VM (P = 0.028) and 20.8% in the ST (P = 0.037, Figure 6C). Decreased phosphorylation in the linker region might be indicative of weaker modulatory intracellular signalling. However, there is no indication of any significant compensatory up-regulation in activity at the Smad2 carboxy-terminal.

### Cross-talk between Smad3 and Erk1/2, but not JNK or p38 MAPKs

**Figure 7:**
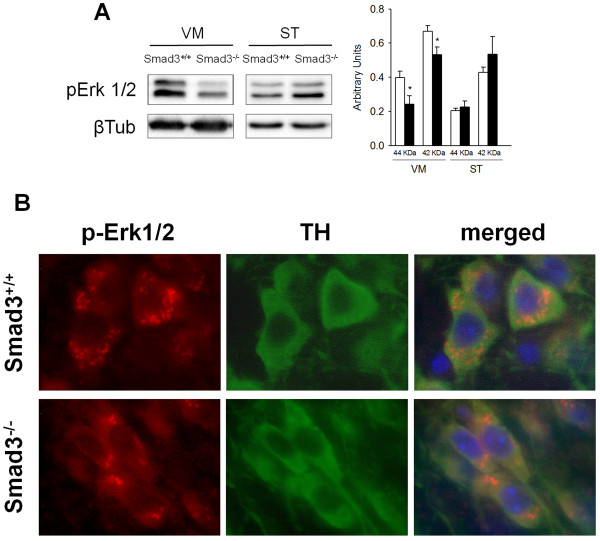
**Decreased Erk1/2 signalling in dopaminergic neurons of Smad3^-/- ^mutants**. (A) Analysis of the phosphorylation state of Erk1/2 MAPK in the VM and ST of Smad3 null mice identifies a specific decrease in Erk1/2 activation in the VM (*P < 0.05, Student's *t-*test, n = 6 per genotype, each measured two to three times). (B) Immunolabelling for p-Erk1/2 defines granular cytoplasmic structures in dopaminergic neurons that are fewer when Smad3 is depleted.

The decreased activation in the linker region of Smad2 may imply a deficiency in MAPK signalling. Extensive cross-talk between TGF-β signalling and JNK, Erk or p38 occurs at different levels to promote or inhibit apoptosis [11]. Furthermore, JNK-cJun activation has been implicated in the pathogenesis of PD [25]. In order to determine whether an interaction between Smad3 and MAPKs exists in the nigrostriatal system, we evaluated the endogenous levels of phosphorylated JNK, p38 and Erk1/2 in Smad3 null mice. The phosphorylation of JNK and p38 in the VM and ST was similar in Smad3^+/+ ^and Smad3^-/- ^mice (Additional file 6A-B), as were the striatal levels of p-Erk1/2 (P = 0.537, for p-ERK2^p42^, Figure 7A). However, Smad3 ablation led to a significant decrease in p-Erk1^p44 ^(38.7%, P = 0.032) and p-Erk2^p42 ^(20.3%, P = 0.038) in the VM (Figure 7A). Immunohistochemistry for p-Erk1/2 showed a granular distribution in dopaminergic neurons of the SN. Interestingly, a general decrease in the number of these cytoplasmic granules labelled for p-Erk1/2 was detected in Smad3 null mice (Figure 7B, data not shown). In summary, two MAPKs implicated in the induction of cell death, such as JNK and p38, are not regulated by Smad3 under basal conditions. However, activation of the pro-survival MAPK Erk1/2 was diminished in nigral dopaminergic neurons after Smad3 depletion. These results suggest that Smad3 activates Erk1/2 in dopaminergic neurons and that Smad3 deficiency may decrease the trophic support provided by Erk1/2 signalling.

### Increased expression and aggregated α-synuclein in Smad3^-/- ^mice

**Figure 8:**
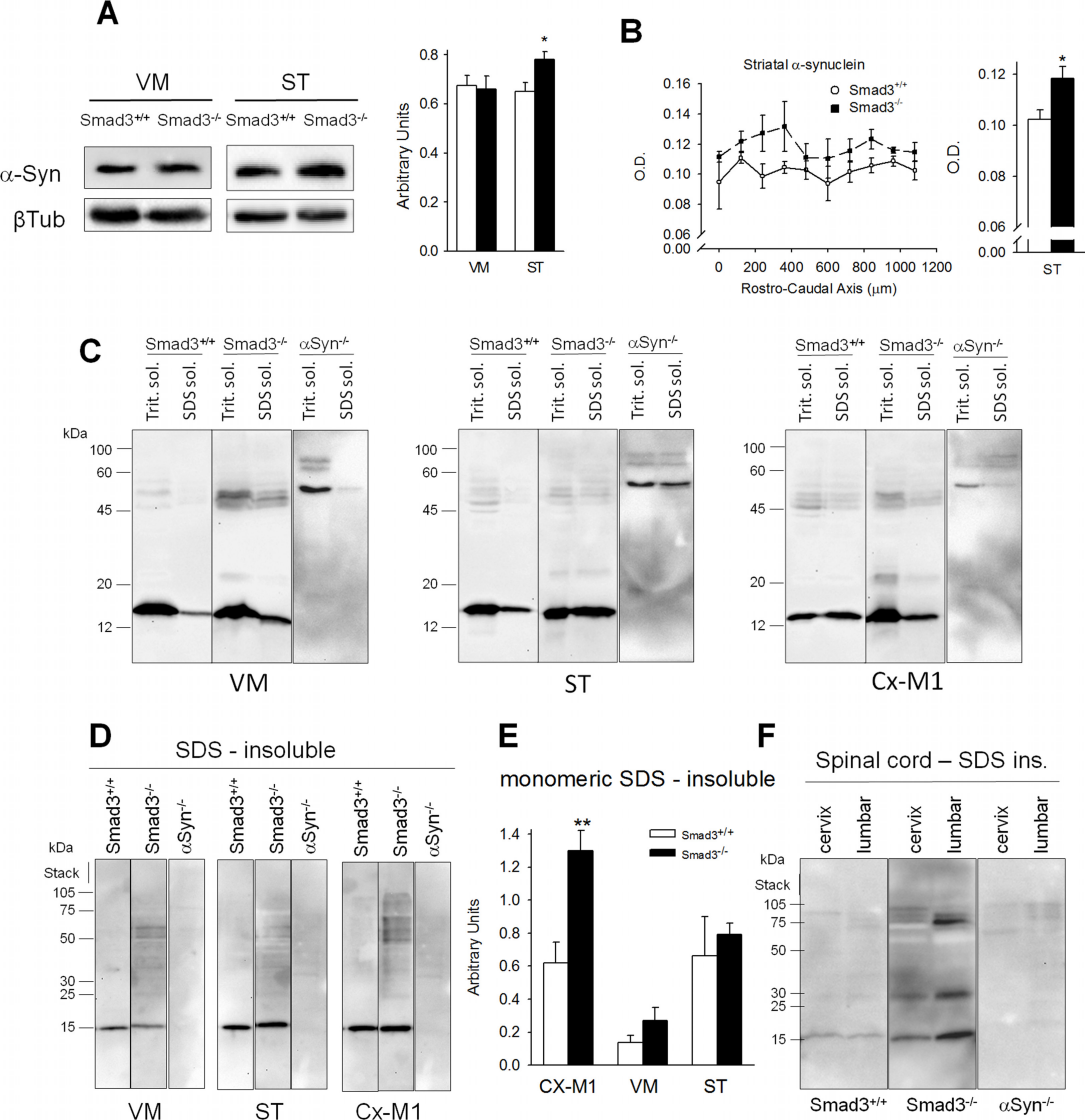
**Increased expression and aggregated α-synuclein in Smad3^-/- ^mice**. (A) Immunoblots of ST and VM tissue show that Smad3 deficiency increases the striatal α-synuclein content (*P < 0.05, Student's *t*-test with n = 6 mice per genotype, measured in triplicate). (B) Further evaluation of α-synuclein expression by immunohistochemistry along the rostro-caudal axis of the ST. Twenty brain sections per mice were counted from Smad3^+/+ ^(n = 5) and Smad3^-/- ^(n = 6). Figure indicates values for the rostral 1000 μm. O.D. quantification shows an increase in the ST of Smad3 null mice (*P < 0.05, Student's *t*-test). (C-F) Several nervous system regions from Smad3 wild-type and null mice were subjected to graded detergent extraction (Triton-soluble, SDS-soluble, and SDS-insoluble fractions) and analyzed in Western blots. The molecular weight markers on the left indicate the protein size in kilodaltons (**P < 0.01, Student *t-*test, n = 3 for Smad3^+/+^, n = 5 for Smad3^-/-^, n = 2 for α-synuclein^-/- ^mice).

**Figure 9:**
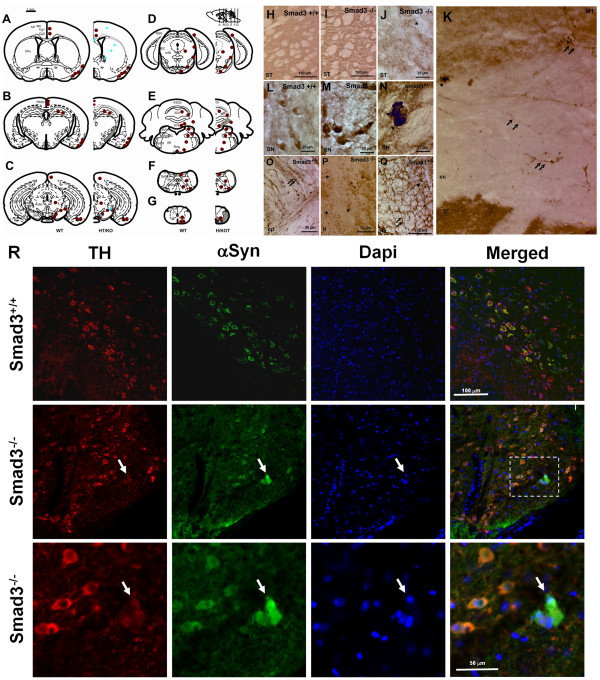
**Aggregation of α-synuclein in specific brain areas**. (A-G) Rostrocaudal distribution of α-synuclein in Smad3 wild-tupe and deficient mice. Red points represent positive α-synuclein staining in wild-type and null mutant. Blue asterisks illustrate cellular and neuritic aggregates present in old heterozygous and young Smad3 null mice. (H-Q) Photomicrography showing α-synuclein positive structures. (H-J) ST striatum, (K) M1 primary motor cortes, cc corpus callosum in Smad3^-/-^, (L-N) SN substantia nigra, (O) cp cerebral peduncle, (P) ll lateral lemniscus, and (Q) fa, anterior funiculus of the spinal cord. The double arrows show positive fibres detected in Smad3 deficient mice, asterisks point to synaptic terminals with a punctuate morphology, and the arrowheads indicate perykarial inclusions. (R) Confocal microscopic images of TH/α-synuclein/Dapi triple labelling in the SN of Smad3^+/+ ^and Smad3^-/- ^mice. In control tissues, α-synuclein is present in the cytoplasm of dopaminergic neurons. The aggregates observed in Smad3 deficiency are double-labelled for both molecules (arrow). The window is further magnified to get a better appraisal of the accumulation of α-synuclein in a TH(+) neuron.

The molecular events detected in Smad3^-/- ^mice were highly suggestive of early signs of parkinsonism. One of the pathological hallmarks in PD is abnormal α-synuclein aggregation and inclusion body formation. When we examined the expression of α-synuclein in immunoblots of the VM and ST of these mice, similar protein levels were found in the VM (Figure 8A). However, Smad3 deficiency increased the expression of α-synuclein in the ST by 20% (P = 0.028). To further assess this response, we performed immunohistochemistry in the ST and we quantified the optical density of α-synuclein fibres in this area (Figure 9H-J). Smad3^-/- ^mice displayed a 16% trend to increase (P = 0.024) in the density of α-synuclein along the rostrocaudal ST when compared to wild-type mice (Figure 8B).

**Figure 10:**
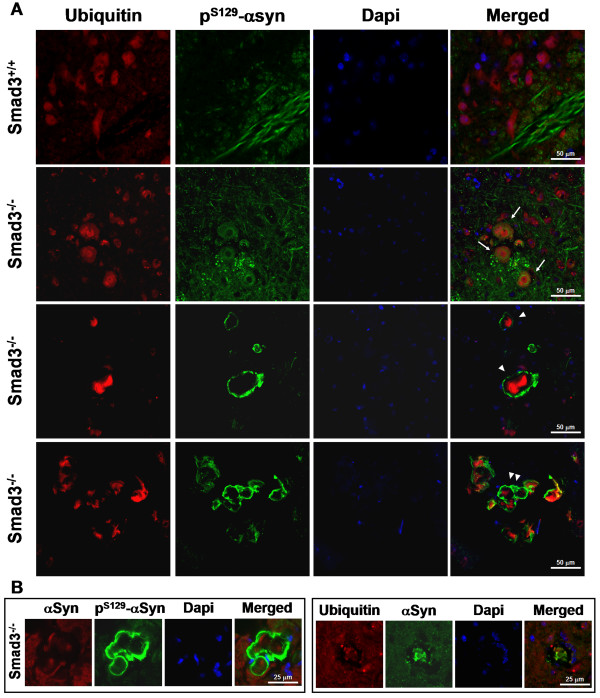
**α-synuclein deposits in Smad3 deficiency are ubiquitinated and S129 phosphorylated**. (A) Confocal images of aggregates double-labelled with ubiquitin and P^S129^-α-synuclein in the ventral midbrain of Smad3 deficient mice. Different morphologies could be detected, from a clear core of ubiquitin surrounded by a halo of P^S129^-α-synuclein (arrows), to strong accumulation of ubiquitin and P^S129^-α-synuclein in isolated (arrowhead) or groups (double arrowhead) of deposits. (B) α-synuclein staining co-localize with ubiquitin within the core of the aggregates.

Biochemically, α-synuclein inclusions are resistant to extraction with non-ionic detergents like Triton X-100. Conversely, extraction with ionic detergents such as SDS disrupts α-synuclein aggregation into monomers and SDS-stable oligomers [26]. We detected aggregated forms of α-synuclein by immunohistochemistry (see below, Figures 9K, N, R and 10). A range of aggregates is observed, from strongly- to mildly-detected, in male Smad3 deficient mice at this age (2-3 months old). We further characterized these aggregates by sequentially extracting tissue from the VM, ST and M1 motor cortex in detergents of increasing strength [27]. To verify the specificity of the signal detected, α-synuclein null mice were used as a control. In Western blots, a specific 12-14 kDa band that corresponded to α-synuclein monomers was detected, which was not detected in the α-synuclein^-/- ^mice. Indeed, most α-synuclein monomers were extracted in the Triton X-100 and SDS soluble fractions (Figure 8C). It should be noted that the antibody reacts with non-specific bands in the α-synuclein^-/- ^mice that differ from those observed in mice of the Smad3 colony. Interestingly, soluble oligomers of 45-50 kDa could be detected in the Triton X-100 phase of VM from Smad3 null mice and a faint 22-24 KD band was detected in Smad3^-/- ^mice, mainly present in the primary motor cortex, suggestive of soluble dimers. High molecular weight SDS-insoluble aggregates were detected in the VM and motor cortex of Smad3 null mice (Figure 8D). Quantification of insoluble monomeric α-synuclein highlighted a 2.1-fold induction (p = 0.010) in the motor cortex in Smad3 null mice (Figure 8E). Interestingly, there was a clear increment of SDS-insoluble monomers and oligomers in the spinal cord of Smad3 null mice, primarily in the lumbar region (Figure 8F), suggesting the appearance of inclusions in these regions.

We further characterized α-synuclein by immunohistochemistry along the rostrocaudal brain axis, and we detected α-synuclein aggregates in neurites and cell bodies of different telencephalic, mesencephalic and romboencephalic regions (Figure 9 A-Q). Indeed, the specificity of this staining was verified in α-synuclein null mice (data not shown). To further study the distribution of α-synuclein, we compared old mice (21 month-old wild-type and 19 month-old heterozygous for Smad3), as well as young mice (3 month-old wild-type and Smad3 null mice). There was no difference in the distribution of α-synuclein between young Smad3 heterozygous and wild-type mice. However, α-synuclein aggregates where detected in young Smad3 null mice and in old Smad3 heterozygous mice, suggesting a gene dosage and age dependent progressive effect. Interestingly α-synuclein aggregates were detected in fibres of the primary and secondary motor cortex, cingulate cortex, ST and corpus callosum (Figure 9A-D, H-K). Moreover, perinuclear α-synuclein aggregates were evident in the cell bodies of the SN, paralemniscal nucleus, and pontine nuclei of old heterozygous and primarily young Smad3 null mice (Figure 9L-N). Furthermore, Smad3 deficient mice manifested a clear increase in staining, associated with an irregular morphology and neurite thickness, in fibres from the internal capsule, corpus callosum, lateral lemniscus, cerebral peduncle and spinal trigeminal tract (Figure 9A-E, O-P). At the level of the 2^nd ^and 3^rd ^cerebellar lobe (Figure 9E), and of the anterior white matter of the spinal cord (Figure 9G, Q) positively labelled cells resembling glia were detected (star shape appearance, arborizing processes, and a mean diameter of 4.3 ± 0.5 μm) [28], together with positive fibres. In the lateral lemniscus, a broad dentritic arbor was detected (Figure 9P).

In order to verify that the α-synuclein aggregates detected in the SN of Smad3 deficient mice are degenerating dopaminergic neurons, we performed confocal imaging of α-synuclein and TH double-labelled neurons (Figure 9R). Co-localization of TH and α-synuclein is observed in the cytoplasm of dopaminergic neurons of the SN, both in control and Smad3 deficient mice. Furthermore, α-synuclein aggregates present in Smad3 deficiency co-localize with TH in this brain area, further suggesting a relationship between the dopaminergic alterations detected in Smad3 deficiency and the presence of α-synuclein aggregates.

### α-Synuclein deposits in Smad3 deficiency accumulates ubiquitin and P^S129^-α-synuclein

Phosphorylation of serine 129 is a widespread α-synuclein post-translational modification within LB and inclusions isolated from human brain with PD, Multiple System Atrophy (MSA), dementia with Lewy bodies (DLB) and other synucleinopathies. Furthermore, the majority of α-synuclein found in LB are mono- to tri-ubiquitinated [29]. In order to evaluate whether these post-translational modifications of α-synuclein are present in the aggregated forms detected in Smad3 deficiency, we performed double-immunostaining using anti-P^S129^-α-synuclein and anti-ubiquitin antibodies in the SN of Smad3 deficient mice (Figure 10A). In the wild-type mice ubiquitin could be found in the cytoplasm of neurons and P^S129^-α-synuclein in neurites and dendrites. However, in Smad3 deficient mice we could detect aggregates with both proteins. Interestingly, in mice with less clear deposits of α-synuclein the distribution of ubiquitin and P^S129^-α-synuclein revealed a core of ubiquitin, surrounded by a halo of P^S129^-α-synuclein (Figure 10A, arrows). It is of note that accumulation of ubiquitin could also be observed within these halos. On the other hand, mice with more clear inclusions detected by anti-α-synuclein antibody show stronger accumulation of ubiquitin, surrounded by a strong rim of P^S129^-α-synuclein. These ubiquitin/P^S129^-α-synuclein double-labelled aggregates were found isolated (arrowhead) or in small groups (double arrowhead). Double-labelling with α-synuclein antibody shows co-localization with ubiquitin within the core of these deposits (Figure 10B).

This core/rim cellular distribution may resemble those observed in human patients with LB diseases [29] and further suggest a key role of Smad3 signalling in the homeostasis of α-synuclein.

The presence of α-synuclein inclusions, with post-translational modifications observed in the human LB, in specific brain areas of Smad3 deficient mice suggests a neuropathological status in these mice, which together with the strong DA catabolism, the loss of dopaminergic neurons and decreased trophic and astroglial support detected, may resemble early symptoms of parkinsonism in these mice.

## Discussion

**Figure 11:**
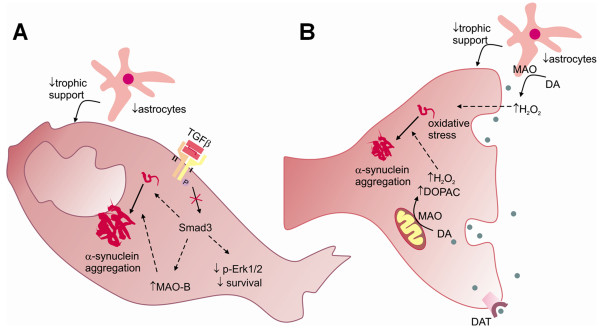
**Illustration of the working model of pathogenic mechanisms of Smad3 deficient mice, in the cell body (A) and synaptic terminal (B) of mesostriatal dopaminergic neurons**.

The strong catabolism of striatal DA associated with the increment of α-synuclein expression and inclusions in selected brain areas, which are phosphorylated at Ser^129 ^and ubiquinated, might represent an interesting model of parkinsonism and/or α-synucleinopathies. Moreover, Smad3 knockout mice have fewer astrocytes in the SN, less nigral dopaminergic neurons, diminished trophic support mediated by Erk1/2 signalling, and increased oxidative stress (Figure 11).

In the mouse brain, TGF-β ligands, receptors and Smad signalling molecules are expressed in the VM and ST. Indeed, the dopaminergic neurons of the SN express Smad3, suggesting that TGF-β ligands can act directly on these neurons. The loss of dopaminergic neurons in Smad3^-/- ^mice from the rostral SN might result from an alteration during the perinatal stage due to TGF-β3 signalling [15]. However, adult Smad3^-/- ^mice do not manifest the overt DA neuron loss observed in TGF-β3 null mice, resembling the effect of TGF-β1 deficiency. Moreover, the decrease in the number of TH-ir neurons in the rostral SN is not observed at P0, suggesting no developmental defect but a postnatal degenerative process. An alternative pathway could account for TGF-β3 during perinatal stage, such as those involving Smad2. Moreover, mRNA TGF-β3 is not present in the adult VM, and several ligands should be considered to activate Smad3 in the adulthood, such as TGF-β1, -β2, Inhibin, Activin, or GDF1. Our results suggest that Smad3 deficiency may be particularly important for adult midbrain pathology since we detect a progressive loss of dopaminergic neurons and a progressive onset of α-synuclein aggregation, with a late onset in the heterozygous mice (19-20 months), when compared with the early onset in the null mice (2-3 months). These neurons may be compromised by decreased Erk1/2 trophic support and increased striatal oxidative stress. It has already been established that Erk1/2 can phosphorylate Smad3 in the linker region, and Erk1/2 and Smad3 converge on the promoters of TGF-β targeted genes [30]. On the other hand, TGF-β can activate Ras-Erk1/2 signalling in a Smad-independent manner [31]. However, our studies show another level of interaction, because Erk1/2 activity is dependent on Smad3 in dopaminergic neurons. Erk1/2 signalling has been implicated in numerous biological processes such as cell survival and neurite outgrowth. Erk1/2 can be compartmentalized to different subcellular structures, such as the nucleus, Golgi, endosomes or plasma membrane. Notably, phosphorylated Erk1/2 has been detected in PD within SN neurons, and with a granular distribution in mitochondria and autophagosomes [32].

Primary cultures of astrocytes derived from human and mouse brain are known to secrete TGFβ molecules and to express the Smad3 signalling pathway. In vitro studies have shown the role of TGFβ/Smad3 in the secretion of TGF-β2 for the increment of extracellular matrix proteins in human optic nerve head astrocytes, in the context of glaucoma [33], the regulation of iNOS production by TGF-β1/Smad3 after inflammation [34], or the induction of Jagged1-Notck signalling for oligodendrocyte progenitor proliferation and differentiation [35]. Interestingly, TGF-β1 arrests astrocyte proliferation in G1 phase, through the induction of p15^INK4B ^[36]. Hence, we could expect an increase in the number of astrocytes in Smad3 deficiency. However, we observe a decrease in the number of astrocytes in the SN and striatal matrix of these mice. Previous studies from Wang and colleagues [36], using astrocyte cultures derived from Smad3 null mice, have shown that Smad3 is necessary, but not sufficient, for the induction of p15^INK4B ^and growth inhibition, suggesting an indirect mechanism for Smad3 on astrocyte proliferation. On the other hand, TGF-β1 is normally expressed at low levels in the basal state of mice and this mechanism on astrocyte proliferation could be only active after brain injury [3-10]. Our results suggest a protective role of Smad3 signalling on astrocytes in the basal state. Astrocytes provide homeostatic control of the extracellular environment of neurons. The number of astrocytes decreases in the SN and dorsal striatal matrix of Smad3 null mice and hence, DA neurons and their striatal terminals may have fewer surrounding astroglial cells to detoxify oxygen free radicals and to secrete neurotrophic factors. Furthermore, astrocytes in the ST may have atrophy in their arborization, with thin and elongated processes, suggesting a phenotypic alteration in the remaining astrocytes. This limited astroglial environment might make neurons more vulnerable to oxidative stress and provide less trophic support to DA neurons. Indeed, loss of astrocytes and dystrophic astrocytes has been associated to several dementias, which correlates with the severity of the disease. In this sense, it is suggested that atrophy of astroglial cells may develop before gross neurodegenerative alterations [37].

Although Smad3 does not regulate striatal TH protein content or DA levels, DA turnover is doubled in Smad3 knockout mice, primarily due to MAO-dependent catabolism. This neurotransmitter degradation might be specific for DA, because striatal serotonin metabolism is not altered. DA turnover is elevated in the brain of PD patients and in experimental models after partial loss of dopaminergic neurons. Partial neuronal degeneration leads to over-activity of the remaining dopaminergic neurons, inducing increased DA release and turnover in order to maintain both normal DA levels in the synaptic cleft and dopaminergic input to postsynaptic targets. However, this mechanism of compensation may contribute to the progress of the disease by promoting endogenous oxidative stress [38]. Increased MAO-catalyzed H_2_O_2 _production can also damage mitochondria through the oxidation of GSH [21]. A similar situation is observed after Smad3 ablation. Indeed, the increase in DA turnover in the ST of Smad3 deficient mice increments the production of H_2_O_2 _and oxidative stress, as suggested by the increment in GSH levels, in an attempt to detoxify cells. Moreover, the 35.9% increment in GSH levels does not seem to be sufficient to detoxify the 2-fold induction in H_2_O_2 _and over time, the GSH system could be overwhelmed, leading to extensive oxidative damage.

The increment in DA catabolism without any modification in DA levels may be related to alterations in different processes, such as increased MAO activity or decreased vesicular DA storage. An alteration in DA reuptake by synaptic vesicles through the vesicular transporter VMAT-2 would leave the DA in the cytosol, exposed to degradation by MAO. However, we have not detected any variation in VMAT-2 striatal protein content. On the other hand, the increased catabolism of DA appears to be due to MAO-B activity, and its increment could degrade extracellular DA producing less activation in post-synaptic targets. Finally, Smad3 might directly regulate MAO-B at the transcriptional level although this remains to be clarified. The role of MAO-B in the pathogenesis of PD is currently being examined and clinical studies indicate that its inhibition may slow the progression of the disease. Indeed, MAO-B inhibitors are used clinically with L-DOPA treatment with interesting protective effects [39].

One of the major pathological events in PD, and of α-synucleinopathies, is the abnormal aggregation of α-synuclein in specific brain areas. Indeed, increased endogenous α-synuclein level due to gene dosage promotes the disease in humans [40]. In this sense, the increased expression of α-synuclein in the ST of Smad3 null mice is suggestive of a progressive pathological state. Furthermore, the increase in soluble and insoluble monomers/oligomers, the presence of detergent-insoluble oligomers of α-synuclein in the VM, motor cortex and spinal cord, as well as the aggregations in the perikarya and neurites in the nigrostriatal system, are similar to the properties of α-synuclein inclusions in human disease [26,41,42]. Accordingly, the accumulation of ubiquitin and P^S129^-α-synuclein within these deposits, and their cellular distribution as a core/halo, further suggest post-translational modifications similar to that present in LB of human α-synucleinopathies [29]. These results suggest that α-synuclein may become cross-linked, probably as a consequence of increased DA catabolism and oxidative stress. Indeed, 3,4-dihydroxyphenylacetaldehyde (DOPAL), a MAO intermediate metabolite of DA, can induce toxic aggregation of α-synuclein [43]. Alternatively, we could speculate that Smad3 might directly regulate the transcription of the α-synuclein gene. It is already known that α-synuclein can modulate DA metabolism by regulating TH, and by directly binding to DA, VMAT-2 and DAT [44]. In this manner, the increased catabolism in DA observed in Smad3 null mice could be due to the prior increase in α-synuclein expression.

These findings suggest a selective pathology in specific brain regions by Smad3 deficiency. Moreover, late onset of α-synucleinopathies in the heterozygous mice when compared with the early onset in the Smad3 null mice suggests that aggregates accumulate in a progressive and gene dosage dependent manner with age. Indeed, the specific α-synuclein aggregation in the nigrostriatal system, and in the cingulate and motor cortices, is also detected in PD [44-46]. However, alterations (increased staining, irregular morphology, and neurite thickness) in other regions of the brain of Smad3 null mice (pontine nuclei, cerebellar white matter, cerebral peduncle, diencephalic nuclei and internal capsule), as well as the presence of glial cells in the cerebellum and spinal cord may correlate with other synucleinopathies such as Multiple System Atrophy [47]. Though, Smad3 deficiency manifests phenotypic symptoms, such as curvature of the spine (kyphosis) and resting tremor that is characteristic of parkinsonism. The mechanism involved in the formation of inclusions from wild-type human α-synuclein is still unclear, although the evidence that a TGFβ-Smad3 deficiency may induce their formation highlights a new interesting mechanism to be explored.

Mice lacking the α-synuclein, parkin, DJ-1 or PINK1 expression have deregulated striatal DA metabolism, release or reuptake, without any alteration in the number of dopaminergic neurons in the SN. While in six independent parkin deficient mice generated there are no clear nigrostriatal deficits or common phenotypes, increased extracellular DA, decreased DAT, increased GSH levels and differences in the catabolism of DA have been described [48-50]. Neither the number of dopaminergic neurons nor DA striatal levels and turnover are altered in DJ-1 or PINK knock-out mice. However, in these mice the evoked DA overflow diminishes due to increased DA uptake [51] or to the decreased quantal release of DA [52], respectively. α-Synuclein knock-out mice have less striatal DA content, enhanced activity-dependent DA release and reduced synaptic vesicle reserve pools [53,54], which may be due to a uninhibited DA vesicular release [55]. All these mutant mice illustrate that impaired presynaptic DA metabolism, release or reuptake may be common in PD, suggesting that the strong catabolism of DA detected in Smad3 null mice may be an early mechanism for the degeneration of dopaminergic neurons.

## Conclusions

The phenotype observed in Smad3 knock-out mice shows that TGFβ-Smad3 signalling regulates the catabolism of DA, promote the survival of dopaminergic neurons and astrocytes and participate in the metabolism of α-synuclein in specific brain areas. Smad3 deficiency may correlate with early signs of parkinsonism, and alterations in TGFβ-Smad3 signalling may contribute to the pathogenesis of parkinsonism. Indeed, Smad3 null mice could be useful as a model to explore the relationship between inflammation, trophic support, DA turnover and α-synuclein pathologies. Moreover, this model may be valuable to screen for novel therapeutic strategies that inhibit or reverse α-synuclein aggregate formation and that regulate DA catabolism.

## Materials and methods

### Immunohistochemistry

Six male 8 to 14-weeks old mice were used per group. The animals were deeply anesthetized with Avertin and perfused via the ascending aorta with 0.9% saline followed by fresh, ice-cold 4% paraformaldehyde in 0.1M phosphate-buffered saline (PBS), pH 7.4. The animals brains were removed, postfixed at 4°C for 2 h in the same fixative, and cryoprotected in 12% sucrose-PBS solution for 48 h at 4°C. The brains were snap-frozen in liquid dry ice-cooled isopentane, and 20 μm coronal cryostat sections were cut and stored at -80°C until use. For TH/Smad3 co-immunolabelling, amplification of the Smad3 signal was obtained using the TSA™ Plus Cyanine 3 System (PerkinElmer, Massachusetts) with some modifications to reduce background. Tissues were permeabilized for 1 h with PBS plus 1% Triton X-100. Endogenous peroxidase activity was quenched with 0.3% H_2_O_2 _in methanol, and non-specific staining was blocked with 10% normal serum/0.3% Triton X-100 in PBS plus 10 μg/ml avidin. Brain sections were incubated at 4°C overnight in blocking solution plus 50 μg/ml biotin with the mouse anti-TH (1:1000, Chemicon, California) and rabbit anti-Smad3 (1:100, Abcam, Cambridge. UK) primary antibodies. The sections were rinsed in PBS and then incubated with anti-mouse Alexa Fluor 488 (1:150, Molecular Probes, Invitrogen) for 1 h at room temperature. After rinsing in PBS, the sections were incubated with biotinylated anti-rabbit IgG for 1 h, rinsed in PBS, and exposed to an avidin-biotin peroxidase complex for 1 h (Vectastain, VectorLabs, California). After washing in PBS, Cy3-Tyr was diluted 1:200 in the kit amplification solution, and it was added to the sections for 10 min at room temperature. After rinsing in PBS, the brain tissue was mounted in ProLong Gold (Invitrogen) and for quantitative statistical analysis, the images were processed using Image-Pro Plus 4.0 software (MediaCybernetics, Bethesda, MD) and its Pearson's correlation application.

To identify apoptosis in the SN, the ApopTag Peroxidase In Situ Apoptosis Detection kit (Chemicon) was used according to the manufacturer's instructions. Co-labelling of TUNEL(+) cells (visualized with diaminobenzidine as a substrate), and TH(+) neurons (visualized by fluorescence) was performed Briefly, antigen retrieval by boiling slices in 10 mM citrate buffer pH 6.0 for 10 min was followed by inhibition of endogenous peroxidase activity with 3% H_2_O_2_. Sections were incubated in ApopTag equilibration buffer for 15 min at room temperature, and then in working strength TdT enzyme buffer during 1 h at 37°C. After washing, sections were exposed to the anti-digoxigenin peroxidase conjugate overnight at 4°C, and the peroxidase activity was visualized with diaminobenzidine as a substrate. TH labelling was performed in the same sections, using mouse anti-TH (1:300) and anti-mouse Alexa Fluor 488 as previously described. TUNEL positive controls were conducted by treating sections with DNase I (1 μg/ml) to induce DNA strand breakage and as negative controls TdT was excluded from the assay.

Standard chromogenic immunohistochemistry was performed for TH, DAT, GFAP, and α-synuclein [17] using rabbit anti-TH (1:3000, Institut J. Boy, France), rat anti-DAT (1:500, Chemicon), rabbit anti-GFAP (1:2750, Abcam), and mouse anti-α-synuclein antibodies (1:1000, BD Transduction Lab, California). The α-synuclein staining was further developed using the Vector MOM™ peroxidase kit (Vector Labs). Measurement of the cell body (maximum diameter) of α-synuclein positive cells in the white matter of spinal cord was performed directly on the screen of a monitor coupled to the microscope using the Scion Image Beta 4.02 software program (Scion Corp., Maryland, USA). Data were expressed as mean ± s.d. of n = 50 cells.

Double immunofluorescence was performed using mouse anti-GFAP (D1:1500, Chemicon), mouse anti-ubiquitin (D1:100, Chemicon), rabbit anti-P^S129^-α-synuclein (D1:50, Abcam), mouse anti-α-synuclein (D1:150), rabbit anti-ubiquitin (D1:200, Abcam), and rabbit anti-TH (D1:100). Vector MOM™ kit (Vector Labs) was used for the antibodies generated in mouse. The fluorescent-labelled secondary antibodies used were Alexa Fluor 488 and Alexa Fluor 568 (D1:250, Invitrogen) from different species. Furthermore, antigen retrieval was used for ubiquitine/P^S129^-α-synuclein, and α-synuclein/TH double labelling. For each case, sections were immunolabelled in triplicate and images were acquired and analyzed by confocal microscopy (Nikkon C1 plus ECLIPSE Ti-e microscope and a 60X Plan Apo VC OIL objective with NA 1.4).

An estimation of the number of astrocytes in the SN and ST using GFAP-ir was performed with the observer blind to the experimental treatment. The ST was delineated with the 2X objective, dividing the dorsal and ventral tiers (as described in [56]). The number of GFAP-ir cells was performed using the objective at 60X, counting every GFAP-ir cell within the delineated region. GFAP-ir cells were counted in a 1:6 series of sections (20 μm per section, 120 μm apart each), from Bregma 1.54 mm to Bregma -0.46 [57]. Moreover, GFAP-ir cells were classified as located in the striatal striosome or matrix. Only the GFAP-ir cells with a continuous labelling in ≥ 3 ramifications and excluding those present in the border were counted. Positive labelling near vessels was excluded due to the difficulty to distinguish unique cells. Total number of GFAP-ir cells was estimated considering the section sampling fraction (1:6). In the SN we analyzed ten brain sections per mouse. For each section, three rectangular regions were delineated (ventral R1, middle R2, and lateral R3, Additional file 4A), with the objective at 2X. For estimation of the number of GFAP-ir cells an objective 20X was used and the counting regions were maintained equidistant from each other along the SN per section. Data is expressed as GFAP-ir cells per mm^2^.

The optical density (O.D.) of TH, DAT and α-synuclein staining in the ST was assessed with the Image Pro Plus 4.0 image analysis software. Briefly, sections of different groups were stained simultaneously, and every reaction, including DAB chromogenic development, was precisely timed to ensure comparability. Images of coronal sections of the whole ST were visualized using a 2X objective and captured under identical lighting conditions. The measurement system was calibrated with a reference slide and incident/black light, and a total of 21 coronal 1:6 serial sections (20 μm per section, 120 μm apart each) per mouse were analyzed. The ST was delineated and O.D. values were used to express staining intensities.

### Stereological cell counting

TH-ir neurons were counted in a 1:5 series of sections (20 μm per section, 100 μm apart each) from Smad3^-/- ^and Smad3^+/+ ^mice using the optical fractionator method, an unbiased cell counting method that is not affected by either the volume of reference or the size of the counted elements [58]. Neurons were counted twice, with the observer blind to the experimental group. The stereology system consists of an Olympus BX51 microscope fitted with a XYZ motorized computer stage (Prior ProScan, Rockland, MA), a digital microcator (Heidenhain, Schaumburg, IL), and the CAST-2 Stereological software (Olympus, Tokyo). Neurons were counted twice, with the observer blind to the experimental treatment, each section was observed at low magnification (objectives 10X and 2X) and the dopaminergic areas A9, A10 and A8 were delineated according to the anatomical landmarks defined in German et al. [59]. The sampling was performed according to unbiased stereological principles using a 100 × 100 μm step length and a counting frame. Dopaminergic neurons were counted under an oil immersion objective (60X, NA = 1.25, Olympus) with the counting unit established as the nucleolus of TH-ir cells. The total positive cell number (N) was estimated using the equation: N = sumQ^- ^× 1/ssf × 1/asf × 1/tsf, where sumQ^- ^is the total number of neurons counted with the fractionator, ssf is the section sampling fraction, asf is the sampling fraction area, and tsf is the sampling fraction thickness. The reliability of the sampling scheme was confirmed by the calculation of the coefficient of error (CE), which was <0.07. For striatal Nissl-stained sections, four mice per genotype were analyzed. Five coronal sections per mice were counted, starting from Bregma 1.42 mm [57], using the same unbiased optical fractionator method previously described, with the following details: 1:6 series of sections (20 μm per section, 120 μm apart each), sampling with 300 × 300 μm step length, and results with a CE ≤ 0.05. Data is expressed as Nissl(+) cells per mm^2^.

### RNA extraction and RT-PCR

The ST and the VM were isolated from 3 six-month-old control C57Bl/6J mice. Freshly dissected tissue was immediately submerged in RNAlater™ solution (Ambion, Austin, TX) to prevent RNA degradation and total RNA was isolated using a RiboPure™ Kit (Ambion) according to the manufacturer's instruction, and stored at -80°C until use. Equal amounts of RNA and cDNA were used for RT and PCR, respectively, in order to provide similar conditions on each reaction. cDNA was prepared from 500 ng of total RNA with oligo(dT)12-18 primers (Invitrogen, California) using SuperScript™ III Reverse Transcriptase (Invitrogen) in a total volume of 20 μl. The cDNA products were stored at -20°C until they were used as templates for PCR. The oligonucleotides used to detect the expression of TGF-β1, TGF-β2, TGFβ-3, ALK1, ALK2, ALK5, TβR-II, Smad2, Smad3, Smad4 and Smad7 are described in Machida et al. [60]. Thirty-five cycles of PCR were performed, using a mix with 1 μl of cDNA product, 1.5 mM MgCl2, 0.25 mM dNTPs, 0.3 μM of each primer, and 2 U of Taq DNA Polymerase (Applied Biosystems, California) in a total volume of 20 μl. The whole PCR reaction was loaded onto a 1.5% agarose gel.

### Mouse line and genotyping

Smad3 knock-out mice [19] have a 129 × C57Bl/6 genetic background. Heterozygous and homozygous Smad3 deficient mice in a C57Bl/6 background are not fertile (data not shown). To establish the colony, we selected breeders with a 50% contribution of each strain by analyzing 60 microsatellites covering 19 autosomal chromosomes (data not shown). For experiments both wild-type and knockout mice were obtained by intercross breeding of heterozygous mice, and they were characterized by PCR analysis of DNA from tail biopsies, with primers: com, 5'-CTCCAGAGTTAAAAGCGAAGTTCG-3'; wt, 5'-AAAATGCTGCACGGAAGCCAGGTC-3'; and neo, 5'-ATTTGTCACGTCCTGCACGACG-3'. The wt and mutant PCR products are 489 bp and 347 bp, respectively. For all analyses, 2-3 month old mice were used as the mortality rate increases in older knock-out mice. Procedures with mice were in accordance with EU and Spanish legislation on animal welfare.

### Monoamine and metabolite analysis

Six animals per group were anesthetized with isofluorane and their striata were rapidly dissected, frozen in dry ice and stored at -80°C until analysis. For catechol analysis, the tissue was homogenized by sonication (VibraCell, level 2 for 30 seconds) in 8 volumes (w/v) of ice-cold 0.4 N perchloric acid (PCA) with 0.5 mM Na_2_S_2_O_5 _and 2% EDTA, and then centrifuged for 20 min at 11,000 g and 4°C. The supernatant was then used to determine the monoamines and their metabolites (20 μl) as well as for glutathione, and the pellet was used for protein extraction. DA and its metabolites DOPAC, HVA, and 3-MT, and serotonin (5-HT) and its metabolite 5-hydroxy-indole-acetic acid (5-HIAA), were measured according to Mena et al., [61]. Briefly, the supernatants were analyzed on a Nucleosil 5C18 column using electrochemical detection (ESA Coulochem, Chelmsford, MA). The mobile phase consisted of a 0.1 M citrate/acetate buffer pH 3.9 with 10% methanol, 1M EDTA and 1.2 heptane sulfonic acid. The detector voltage conditions were D1 (+0.05), D2 (-0.39) and the guard cell (+0.40).

### Glutathione measurements

Total glutathione levels were measured by the method of Tietze [62]. The striatal homogenate (40 μl) used for High-performance liquid chromatography (HPLC) analysis (in 0.4N PCA) was neutralized with 4 volumes of phosphate buffer (0.2 M NaH_2_PO_4_, 0.2 M Na_2_HPO_4_, 0.5 M EDTA, pH 7.5) and the resulting preparation (50 μl) was diluted 1/10 with PBS and mixed with 0.6 mM DTNB, 0.2 mM NADPH and 1U GSH reductase. The reaction was monitored in a P96 automatic microtiter reader at 412 nm for 6 min. Oxidized glutathione (GSSG) was measured by the method of Griffith [63], whereby the neutralized striatal homogenate (120 μl) was mixed with 2-vinylpyridine (1.2 μL) at 22-24°C for 1 h and the reaction was carried out as described above. Reduced glutathione (GSH) was obtained by subtracting oxidized glutathione from total glutathione levels.

### Protein extraction and immunoblotting

The pellet obtained from the striatal homogenate (in PCA) was neutralized with 1/9 (w/v) lysis buffer (0.75% Na_2_CO_3_, 2% SDS, 10 μg/ml leupeptin, 2 μg/ml aprotinin, 1 mM PMSF, 2 mM o-vanadate), sonicated and centrifuged for 30 min at 11,000 g and 4°C. The supernatant was used for protein quantification by the BCA assay (Pierce, Rockford, IL) and 10-20 μg of each sample was resolved by 12% SDS-PAGE for immunoblotting (Bio-Rad, California). Proteins were transferred to PVDF membranes (Hybond-P, Amersham, UK) and detected using a chemiluminescence detection system. The primary antibodies used were raised against TH (1:6000, Institut Jacques Boy, France), p-JNK (1:5000, Cell Signaling, Danvers, MA), p-Erk1/2 (1:3000, Cell Signaling), p-P38 (1:3000, Cell Signaling), p-Smad2-Cter (1:4000, Cell Signaling), p-Smad2-Linker (1:4000, Cell Signaling), β-tubulin (1:10000, Cell Signaling), MAO-B (1:5000, Santa Cruz), VMAT-2 (1:3000, Santa Cruz), α-synuclein (1:6000, BD Transduction Laboratories). Briefly, membranes were blocked for 1 h with 0.4% ECL Advance blocking reagent (Amersham) in Tris-buffered saline (TBS) with 0.1% Tween-20 (TTBS), and incubated with the primary antibody at 4°C overnight. After washing, the membranes they were incubated for 2 h with anti-rabbit IgG-HRP (1:15000, Amersham) or anti-goat IgG-HRP (1:15000, Santa Cruz) in blocking buffer. After rinsing in TTBS, the reaction was visualized using the ECL Advance Western Blotting Detection Kit (Amersham). Images were captured using VersaDoc Imaging System (Model 4000, Bio-Rad), and the band densities were quantified with ImageQuant TL software (Amersham). Band intensities within the linear range were normalized to the signal obtained in the same lane with an anti-β tubulin antibody to control for variability in protein loading. We measured β-tubulin protein levels in Smad3^+/+ ^and Smad3^-/- ^mice (6 mice per group, measured in 6 replicates). No difference in the expression of β-Tub was detected between mice (P = 0.264, data not shown), validating the utility of this protein as a loading control. Each protein was analyzed in 6 mice per group and experiments were performed in duplicate or triplicate.

### Sequential detergent extraction of α-synuclein

The motor cortex, ST, VM, cervical and lumbar spinal cord from Smad3^+/+ ^and Smad3^-/- ^mice (3-4 months old) were dissected out, weighed and homogenized by sonication in 3 ml/g of buffer A (10 mM Tris-HCl [pH 7.6], 0.15 M NaCl, 1% Triton X-100, and protease inhibitors). The α-synuclein null mice were used as a control of the signal (a kind gift from Dr. Isabel Fariñas). Sequential detergent extraction was performed as described by Ihara, et al. [27]. Briefly, the supernatant recovered after centrifugation at 15,000 × g at 4°C for 30 min was considered as the "Triton soluble" extract. The pellet was sonicated and re-extracted with 1 ml/g of buffer B (10 mM Tris-HCl [pH 8.0], 0.15 M NaCl, 1% Triton X-100, 0.5 sodium deoxycholate, and 0.1% SDS), and it was fractionated by centrifugation at 15,000 × g at 4°C for 30 min. This supernatant was considered as the "SDS-soluble fraction". The pellet was again dissolved by sonication and boiled in buffer C (3% SDS and 5% β-mercaptoethanol, 1 ml/g pellet), and the lysate was considered as the "SDS-insoluble fraction" (Ihara, et al., 2007). Protein quantification of each fraction was performed by the BCA (bicinchoninic acid) protein assay, and 50-75 μg of each sample was resolved by 12% SDS-PAGE as described previously.

### Statistical analyses

SigmaStat software (SSPS, Chicago, IL) was used for all analyses and all values were expressed as the means ± s.e.m. The normal distribution of the data was assessed to use parametric or non-parametric tests. Differences between the means were analyzed with the unpaired *t*-test (for normally distributed populations) and Mann-Whitney rank sum test (as non-parametric test). In all analyses, the null hypothesis was rejected at the 0.05 level. (*), (**), and (***) indicates P ≤ 0.05, P ≤ 0.01, and P ≤ 0.001, respectively.

## List of abbreviations

DOPAC: 3,4-dihydroxyphenylacetic acid; 3-MT: 3-methoxy-tyramine; 5-HIAA: 5-hydroxy-indole-acetic acid; COMT: catechol-O-methyl transferase; DLB: dementia with Lewy bodies; DA: dopamine; DAT: dopamine transporter; GFAP: glial fibrillary acidic protein; HVA: homovanillic acid; LB: Lewy bodies; MAO: monoamine oxidase; MSA: multiple system atrophy; O.D.: optical density; PD: Parkinson's disease; 5-HT: serotonin; ST: striatum; SN: substantia nigra; TGF-β1: transforming growth factor-β1; TH: tyrosine hydroxylase; VM: ventral midbrain; VMAT-2: vesicular monoamine transporter.

## Competing interests

The authors declare that they have no competing interests.

## Authors' contributions

STG. RGP, MIC, MJC, MAM, and ASC performed research; XFW contributed new reagents; STG, RGP, MIC and ASC analyzed data; ASC designed research and wrote the manuscript. All authors read and approved the final manuscript.

## Supplementary Material

Additional file 1 **Normal gross morphology of the SN in Smad3 deficiency**. (A) Coronal brain section of a Smad3^+/+ ^and Smad3^-/- ^mouse showing dopaminergic neurons in the SN and the ventral tegmental area stained with an antibody against TH. The mesencephalic gross morphology is not altered in Smad3 deficiency (B) Quantification of TUNEL (+) cells in the SN showed no clear increase on apoptosis in Smad3 deficient mice, (3.60 ± 0.81 in Smad3^+/+^, 4.71 ± 1.57 in Smad3^-/- ^mice, P = 0.590, unpaired *t*-test, n = 6-7 per group). Representative images of the apoptotic cells counted. The criteria to count apoptotic cells was both morphological (cells with nuclear chromatin condensation) and molecular (nuclear DNA fragmentation shown by TUNEL). Only cells with the DNA of the entire nucleus both fragmented and condensed were counted (stage 2 of apoptotic degradation) [20]. These apoptotic cells lacked immunoreactivity for TH (not shown). Click here for file

Additional file 2 **No alteration in striatal TH content in Smad3 null mice**. (A) TH-ir quantification of Smad3^+/+ ^and Smad3^-/- ^mice by O.D. in the dorsal and ventral tiers of the ST. The entire ST along the rostro-caudal axis was counted from Smad3^+/+ ^(n = 5) and Smad3^-/- ^(n = 6) mice. (B) Immunoblot analysis of striatal TH showed no difference between mice. Arbitrary units refers to the TH content normalized to β-tubulin. Bars represent values from duplicate measures of 6 mice per group. Click here for file

Additional file 3 **Trend to decrease striatal DAT expression in Smad3^-/- ^mice**. DAT expression in the rostro-caudal and dorso-ventral axis of the striatum of Smad3^-/- ^mice. Quantification of DAT immunoreactivity (A) in the ST of Smad3^+/+ ^and Smad3^-/- ^mice by optical density (O.D.) in the dorsal (B) and ventral tiers (C) of the ST. For rostro-caudal analyses twenty brain sections per mice were counted from n = 5 mice per genotype. Bar figures indicate segregated values for the rostral (initial 1000 μm) and caudal portions of the striatum. Click here for file

Additional file 4 **GFAP(+)astrocytes in the SN in Smad3 deficiency**. GFAP-ir astrocytes in the SN of wild-type (A) and Smad3 null mice (B). (C) Estimation of the number of GFAP-ir cells in three areas of the SN - ventral (R1), middle (R2) and lateral (R3)- in serial slices (from bregma -3.08 to bregma -3.8). Unpaired t-test, four brain sections per mice from n = 4 mice per genotype. Click here for file

Additional file 5 **Smad3 deficiency does not alter striatal serotonin levels (5-HT) or its catabolite (HIAA)**. Student's t-test, n = 6 for Smad3^+/+ ^and Smad3^-/- ^mice. Click here for file

Additional file 6 **Analysis of the phosphorylation state of JNK and p38 in Smad3 deficiency**. (n = 6 per genotype, each measured two to three times). Click here for file
