# Supplementary figures and images for: Dopamine and α-synuclein dysfunction in Smad3 null mice
Source: Mol Neurodegener. 2011 Oct 13;6:72. doi: 10.1186/1750-1326-6-72 (PMC3219599; doi:10.1186/1750-1326-6-72)

**a**

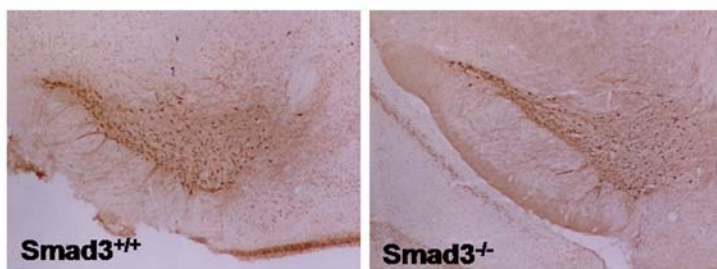

**b**

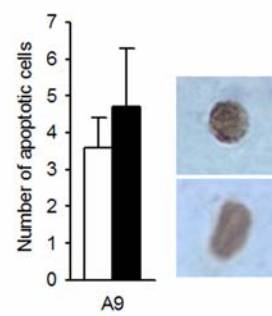

Supplement: Additional file 1 — Normal gross morphology of the SN in Smad3 deficiency. (A) Coronal brain section of a Smad3+/+ and Smad3-/- mouse showing dopaminergic neurons in the SN and the ventral tegmental area stained with an antibody against TH. The mesencephalic gross morphology is not altered in Smad3 deficiency (B) Quantification of TUNEL (+) cells in the SN showed no clear increase on apoptosis in Smad3 deficient mice, (3.60 ± 0.81 in Smad3+/+, 4.71 ± 1.57 in Smad3-/- mice, P = 0.590, unpaired t-test, n = 6-7 per group). Representative images of the apoptotic cells counted. The criteria to count apoptotic cells was both morphological (cells with nuclear chromatin condensation) and molecular (nuclear DNA fragmentation shown by TUNEL). Only cells with the DNA of the entire nucleus both fragmented and condensed were counted (stage 2 of apoptotic degradation) [20]. These apoptotic cells lacked immunoreactivity for TH (not shown). [file 1750-1326-6-72-S1.PDF]

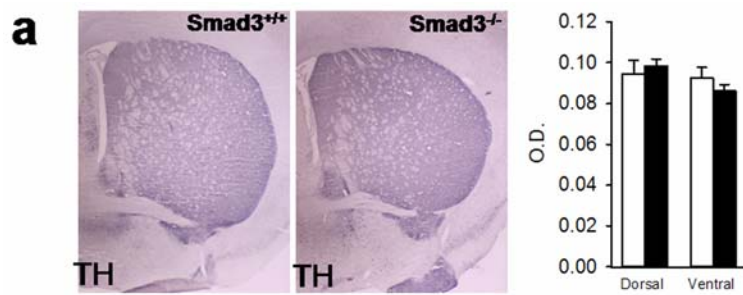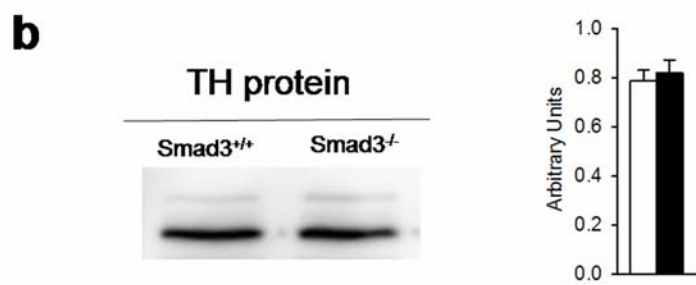

Supplement: Additional file 2 — No alteration in striatal TH content in Smad3 null mice. (A) TH-ir quantification of Smad3+/+ and Smad3-/- mice by O.D. in the dorsal and ventral tiers of the ST. The entire ST along the rostro-caudal axis was counted from Smad3+/+ (n = 5) and Smad3-/- (n = 6) mice. (B) Immunoblot analysis of striatal TH showed no difference between mice. Arbitrary units refers to the TH content normalized to β-tubulin. Bars represent values from duplicate measures of 6 mice per group. [file 1750-1326-6-72-S2.PDF]

**A**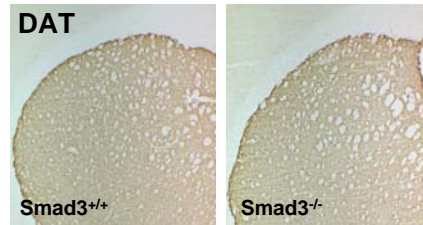**B**

DAT - Dorsal ST

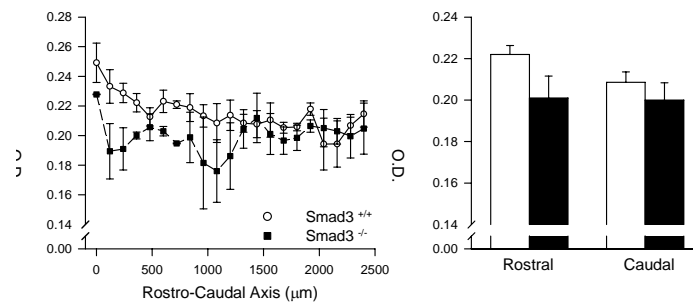**C**

DAT - Ventral ST

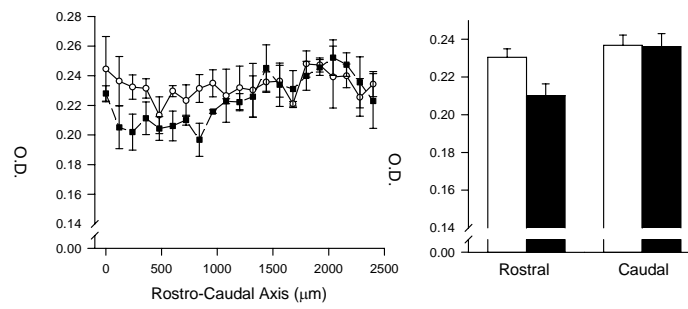

Supplement: Additional file 3 — Trend to decrease striatal DAT expression in Smad3-/- mice. DAT expression in the rostro-caudal and dorso-ventral axis of the striatum of Smad3-/- mice. Quantification of DAT immunoreactivity (A) in the ST of Smad3+/+ and Smad3-/- mice by optical density (O.D.) in the dorsal (B) and ventral tiers (C) of the ST. For rostro-caudal analyses twenty brain sections per mice were counted from n = 5 mice per genotype. Bar figures indicate segregated values for the rostral (initial 1000 μm) and caudal portions of the striatum. [file 1750-1326-6-72-S3.PDF]

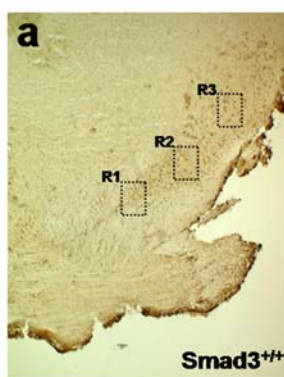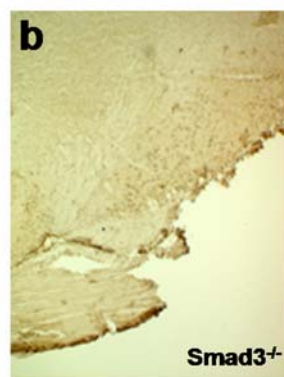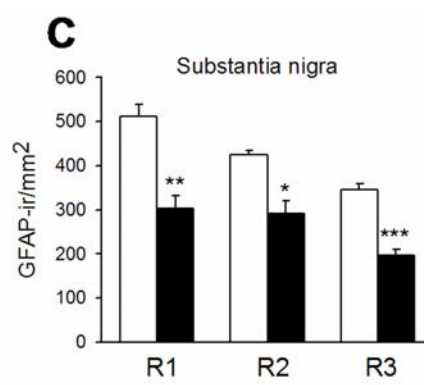

Supplement: Additional file 4 — GFAP(+)astrocytes in the SN in Smad3 deficiency. GFAP-ir astrocytes in the SN of wild-type (A) and Smad3 null mice (B). (C) Estimation of the number of GFAP-ir cells in three areas of the SN - ventral (R1), middle (R2) and lateral (R3)- in serial slices (from bregma -3.08 to bregma -3.8). Unpaired t-test, four brain sections per mice from n = 4 mice per genotype. [file 1750-1326-6-72-S4.PDF]

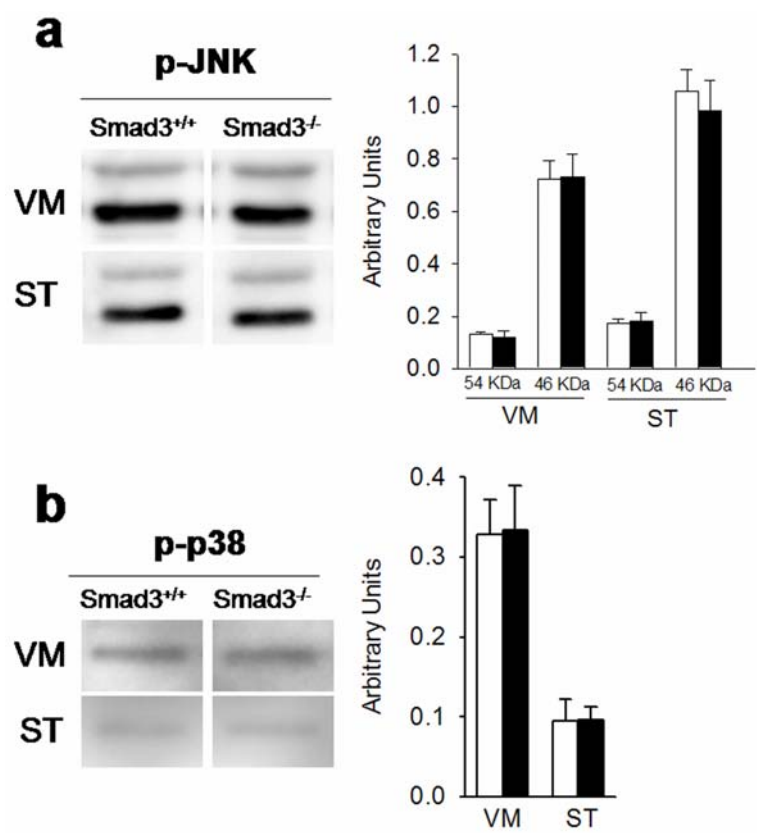

Supplement: Additional file 6 — Analysis of the phosphorylation state of JNK and p38 in Smad3 deficiency. (n = 6 per genotype, each measured two to three times). [file 1750-1326-6-72-S6.PDF]
