# Supplementary material for: Dopamine and α-synuclein dysfunction in Smad3 null mice
Source: Mol Neurodegener. 2011 Oct 13;6:72. doi: 10.1186/1750-1326-6-72 (PMC3219599; doi:10.1186/1750-1326-6-72)
Supplement: Additional file 5 — Smad3 deficiency does not alter striatal serotonin levels (5-HT) or its catabolite (HIAA). Student's t-test, n = 6 for Smad3+/+ and Smad3-/- mice. [file 1750-1326-6-72-S5.PDF]

	$Smad3^{+/+}$ (ng/g tissue)	$Smad3^{-/-}$ (ng/g tissue)
5-HT	865.20 ± 136.12	817.95 ± 32.67
HIAA	574.00 ± 76.63	728.00 ± 41.56
t-test	P=0.945	P=0.108
